# Whole Genome Sequencing of Danish *Staphylococcus argenteus* Reveals a Genetically Diverse Collection with Clear Separation from *Staphylococcus aureus*

**DOI:** 10.3389/fmicb.2017.01512

**Published:** 2017-08-09

**Authors:** Thomas A. Hansen, Mette D. Bartels, Silje V. Høgh, Lone E. Dons, Michael Pedersen, Thøger G. Jensen, Michael Kemp, Marianne N. Skov, Heidi Gumpert, Peder Worning, Henrik Westh

**Affiliations:** ^1^Department of Clinical Microbiology, Hvidovre University Hospital Hvidovre, Denmark; ^2^Department of Clinical Microbiology, Odense University Hospital Odense, Denmark; ^3^Department of Technology, Faculty of Health and Technology, Metropolitan University College Copenhagen, Denmark; ^4^Department of Clinical Microbiology, Herlev University Hospital Herlev, Denmark; ^5^Institute of Clinical Medicine, University of Copenhagen Copenhagen, Denmark

**Keywords:** *S. aureus*, *S. argenteus*, SargPID7903, SargPI, plasmid, phage, *Staphylococcus*

## Abstract

*Staphylococcus argenteus* (*S. argenteus*) is a newly identified *Staphylococcus* species that has been misidentified as *Staphylococcus aureus* (*S. aureus*) and is clinically relevant. We identified 25 *S. argenteus* genomes in our collection of whole genome sequenced *S. aureus*. These genomes were compared to publicly available genomes and a phylogeny revealed seven clusters corresponding to seven clonal complexes. The genome of *S. argenteus* was found to be different from the genome of *S. aureus* and a core genome analysis showed that ~33% of the total gene pool was shared between the two species, at 90% homology level. An assessment of mobile elements shows flow of SCC*mec* cassettes, plasmids, phages, and pathogenicity islands, between *S. argenteus* and *S. aureus*. This dataset emphasizes that *S. argenteus* and *S. aureus* are two separate species that share genetic material.

## Introduction

*Staphylococcus argenteus* (*S. argenteus*) is a newly identified coagulase-positive and catalase-positive *Staphylococcus* species, that previously has been misidentified as *Staphylococcus aureus* (*S. aureus*), given their high phenotypic similarity (Tong et al., 2015). The main phenotypic difference lies in the pigmentation, where *S. argenteus* has non-pigmented colonies due to the lack of the carotenoid pigment, staphyloxanthin, that protects against oxidative stress (Holt et al., 2011). The name *S. argenteus* was proposed in 2015 (Tong et al., 2015) but *S. argenteus* isolates were already described in 2009 in a clinical setting in the Northern Territory of Australia (Ng et al., 2009) with isolates belonging to clonal complex 75 (CC75). More recently, *S. argenteus* isolates belonging to CC1233, CC2198, CC2250, and CC2854 have also been described (Chantratita et al., 2016). *S. argenteus* has been found to be both methicillin susceptible and methicillin resistant, harboring the SCC*mec* IV cassette. Whole genome sequencing has found *S. argenteus* as distant outliers in *S. aureus* phylogenetic trees and an average nucleotide identity of less than 95%, and inferred DNA–DNA hybridization of less than 70% confirms that *S. argenteus* is its own species (Holt et al., 2011). A global distribution is suggested from findings of these bacteria in Australia (Ng et al., 2009), Thailand (Chantratita et al., 2016) and Belgium (Argudín et al., 2016). In a collection of 311 *S. aureus* sepsis samples from Thailand, 58 (18.6%) isolates were found to be methicillin susceptible *S. argenteus*. A study of 1,903 *S. aureus* from Belgium only found 3 *S. argenteus*, two of which were methicillin resistant *S. argenteus* (MRSArg). *S. argenteus* is not generally found in wildlife or domestic animals (Schaumburg et al., 2015; Monecke et al., 2016), except one finding in a wild life African gorilla (Schuster et al., 2016).

In this study, 25 *S. argenteus* isolates from Denmark are described. At the Department of Clinical Microbiology at Hvidovre Hospital, whole genome sequencing (WGS) of methicillin resistant *S. aureus* (MRSA) has been applied as a routine method since 2013 (Bartels et al., 2014). More than 4000 MRSA isolates have been sequenced going back to 2003. We have sporadically whole genome sequenced 17 MRSArg. Furthermore, eight isolates from Odense University Hospital that were suspected to be *nuc*-negative methicillin susceptible *S. aureus* (MSSA) by PCR (Hoegh et al., 2014) turned out to be *S. argenteus* by WGS containing a dissimilar *nuc* gene. This study gives clinical and genomic insight into the distribution of *S. argenteus* in Denmark with a comparison to *globally distributed* genomes.

## Materials and methods

Twenty-five *S. argenteus* isolates from patients in Denmark were identified by WGS. Originally, species identification was done using the MALDI-TOF mass spectra (MALDI Biotyper 3.1, Bruker Daltonics Microflex LT, database MBT DB-5627) from colonies directly transferred to the target plate and isolates were confirmed as MRSA positive using a multiplex real-time PCR assay detecting the presence of *nuc, femA, mecA*, and/or *mecC* or as per Hoegh et al. (2014). Isolates were sequenced as previously described (Bartels et al., 2014) using WGS on the MiSeq system (Illumina, San Diego, CA, USA) using 2 × 150-bp paired-end reads. The reads from the MiSeq were assembled with either Velvet (Zerbino and Birney, 2008) (V1.0.18) in combination with VelvetOptimizer with settings for maximizing N50 or SPAdes (Bankevich et al., 2012)(V3.6.0) using default settings. The assemblies can be found with the accession number PRJEB20633. The multiple sequence alignment was done using mugsy (Angiuoli and Salzberg, 2011) (V1.2.3) with default settings comparing assemblies of the 25 sequenced isolates and 9 assemblies of *S. argenteus* [PRJNA321471, *PRJEB6387, PRJEB6393, PRJEB6394, PRJEB6396, PRJEB6386, PRJNA310972, PRJNA267549* (from human infections), *and PRJEA62885* (from a gorilla)] downloaded from NCBI BioProjects database. Bootstrapping was done using rax *mlHPC* (Stamatakis, 2006). (V7.0.4) with settings –f a –m GTRGAMMA –p 12345 –x 12345 -# 100. Assemblies of these isolates and of *S. aureus* isolates were annotated using Prokka (Seemann, 2014) (V1.11) and the pan-genome calculated using roary with 85, 90, 95, and 100% identity thresholds (Page et al., 2015). To identify antimicrobial resistance genes all assemblies were aligned to the Arg-ANNOT database (Gupta et al., 2014) *using BLASTn* with default settings. Plasmidfinder (Carattoli et al., 2014) was used for plasmid identification. For virulence factor detection the contigs were mapped using BLASTn to the VFDB (Chen et al., 2016). To assess the bacteriophage composition all contigs were screened using Phaster (Arndt et al., 2016). The gene synteny analysis was conducted using genoPlotR (Guy et al., 2011) and the wrapper tool is found at https://github.com/ThomasArn/genoPlotR_wrapper. The *S. argenteus* pathogenicity islands were detected using a homolog search to known *S. aureus* pathogenicity islands (SaPIs). The isolates were compared to other isolates globally distributed, by comparing Sequence Types (ST) defined by Multilocus sequence typing (MLST). For Supplementary Table 1, a search was performed on http://saureus.mlst.net/ and NCBI to identify MLST types and genomes belonging to *S. argenteus*. SCC*mec* types were identified using an in-house script.

## Results

The 25 *S. argenteus* isolates were originally double tested on MALDI-TOF and all had *S. aureus* as the best match (Table 1), but only four isolates had a score above 2.0 for *S. aureus*. There is no *S. argenteus* profile in the MALDI-TOF database. In our collection of 4001 whole genome sequenced MRSA isolates, we found that 17 methicillin resistant isolates were instead *S. argenteus*. This corresponds to 0.35% of our whole genome sequenced MRSA. In addition, eight methicillin susceptible isolates from Odense University Hospital, that were believed to be methicillin susceptible, *nuc* gene negative, *S. aureus* (MSSA), were whole genome sequenced and found to be *S. argenteus*. All *S. argenteus* had a *nuc* length of 670 bp, compared to the normal *S. aureus nuc* length of 687 bp and showed 83% identity with the *S. aureus nuc* gene. The 25 assemblies had a mean of 2,799,643 bp (2,739,502–2,981,617), mean N50 of 99,109 (31,852–266,367) and a mean number of 133 contigs (39–361). All MRSArg carried a SCC*mec*IV cassette. MLST of these strains showed that 17 Danish strains belonged to CC2250, four to CC2596, three to CC1223, and one to CC2854. No Danish strains were found to belong to CC75 or CC2198. (Supplementary Table 1).

**Table 1:** The *S. aureus* MALDI-TOF scores by double testing each *S. argenteus* isolate.

	**MALDI-score < 1.7 = Not reliable identification**	**MALDI-score 1,700–1,999 = Probable genus identification**	**MALDI-score 2,000–2,299 = Secure genus identification, probable species identification**
Number of isolates	3 (H1826, O-6, D7903)	18	4 (O-2, O-9, M5200, M5224)

**Figure 1:**
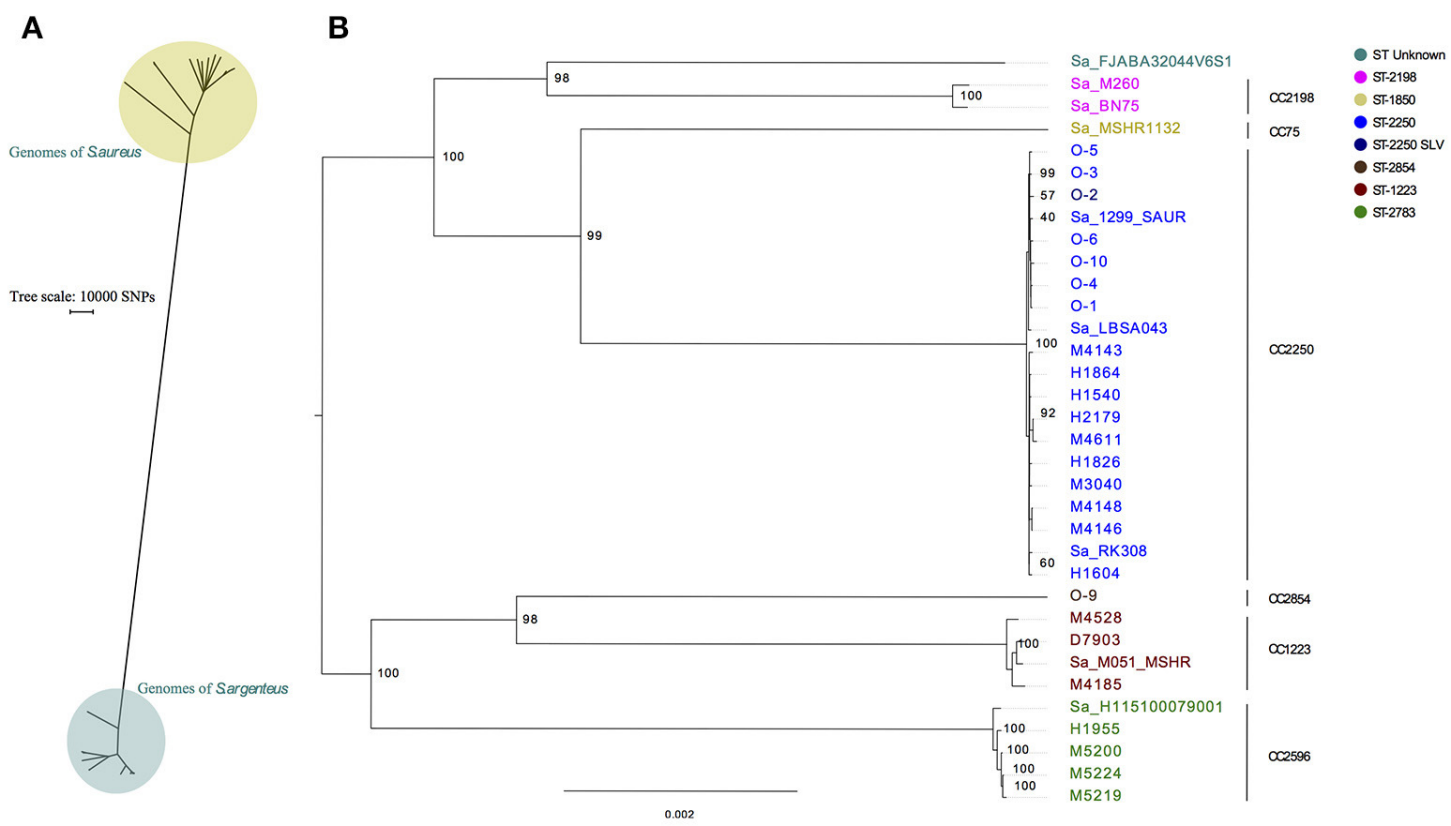
Phylogenetic comparison of *S. argenteus* with *S. aureus* and of *S. argenteus* isolates alone. **(A)** SNP analysis of 34 *S. argenteus* genomes with a representative set of 22 genomes of *S. aureus* all with different sequence types. **(B)** Maximum likelihood phylogenetic tree of *S. argenteus* isolates. The tree is made from multiple sequence alignment of assemblies from 25 isolates from Denmark and 9 references of *S. argenteus* assemblies. One Hundred bootstraps were performed. Prefix of Sa indicates *S. argenteus* reference genomes downloaded from NCBI, O- prefix are Odense isolates and the remaining isolates are from Copenhagen. Bootstrap values are shown at the major breakpoints. The scale bar represents the mean number of nucleotide substitutions per site. The tree is midpoint rooted.

The SNP distance between 22 MRSA with different ST's from the in-house collection and the 25 isolates from this study plus 9 public available *S. argenteus* was identified to be more than 150,000 SNPs and clearly groups *S. argenteus* in its own monophyletic group (Figure 1A). A phylogenetic analysis on the 34 *S. argenteus* genomes was performed and the results clearly showed that all Danish isolates except O-9 (ST2854) clustered together with publicly available *S. argenteus* isolates downloaded from NCBI (Figure 1B).

Furthermore, we saw seven distinct clusters of the isolates where the largest cluster was populated by CC2250 isolates. The isolates H1955, M5219, M5220, and M5224 (all ST2793) are double locus variants of ST2596 and constitute their own cluster. The methicillin susceptible isolates from Odense clustered together, except for O-9 which belonged to ST2854 and was located on a separate branch.

**Figure 2:**
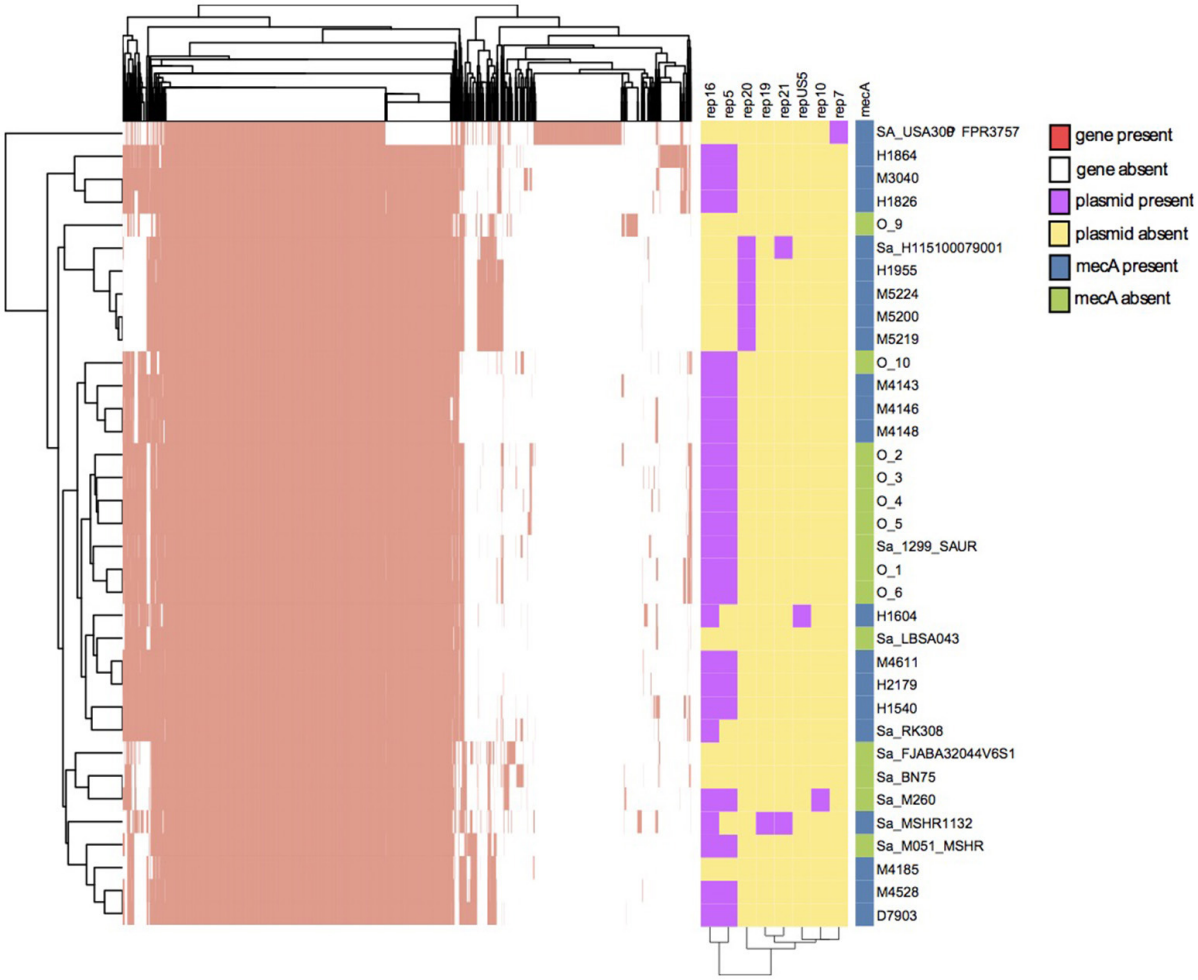
Pan-genome analysis, plasmid identification, and identification of mecA antibiotic resistance genes. The y-axis portrays the isolates. Two dimensional clustering of genes and isolates shows the genes of the pan-genome as present (red) or absent (white) in each isolate with a BLASTp similarity of 90%. The plasmid replicon genes are shown as purple and the absence of replicon gene is shown as yellow. The presence of *mec*A in the isolates is shown to the right. Blue is presence of *mec*A and green denotes absence of *mec*A.

The pan-genome in Figure 2 showed that there are 39% shared genes between the *S. aureus* strain FPR3757 and the 34 *S. argenteus* strains, while the entire *S. argenteus* collection shares a large proportion of their genes. To further evaluate the difference between *S. aureus* and *S. argenteus*, we performed pan-genome analyses at four different BLASTp homology thresholds (85, 90, 95, 100%). The analyses included 22 MRSA genomes, each with different ST's, and the 34 *S. argenteus* genomes. This analysis showed that ~38, 33, 17, and 4% genes were shared between the two species at 85, 90, 95, and 100% homology level, respectively. These gene compositions could be used to discriminate between *S. aureus* and *S. argenteus* at all four thresholds (PCA plot in Supplementary Figure 1).

To identify the antibiotic resistance patterns of the assemblies they were aligned to the Arg-ANNOT database using BLASTn, and we confirmed *mecA* in 17 Danish samples (Figure 2). Other identified antibiotic resistance families included beta-lactamases, macrolide-lincosamide-streptogramin, tetracyclines, and aminoglycosides.

Plasmid replicon identification is shown in Figure 2. Seven plasmid replication genes were discovered in *S. argenteus*. The most prevalent genes were Rep16 and Rep5 found in 24 and 21 isolates, respectively. The replicon gene Rep5 was always identified on the same contig as Rep16, hence these would be considered multi-replicon plasmids. A BLASTn search revealed that all contigs carrying Rep16 gene, had ~99% identity to either of eight plasmids (AP004832.1, BX571858.1, FR821778.1, GQ900392.1, GQ900413.1, GQ900424.1, GQ900467.1, and GQ900478.1), which have all been discovered in either *S. argenteus* or *S. aureus*. A RAST annotation of these plasmids revealed genes with homology to beta-lactamase and cadmium resistance (*CadD* and *CadC*) genes. Furthermore, two isolates (O-2 and O-6) hosted a *CzcD* gene in a Rep16 plasmid. Our collection of *S. aureus* was searched for *CadD* and *CzcD*. *CadD* was found in ~66% of the *S. aureus* isolates and *CzcD* was found in less than 0.3% of the isolates. The length of the plasmid contigs ranged from 3,906 to 23,905 bp and the longest plasmid contigs correspond to the length of the reference plasmid. The three isolates in the CC2596 cluster interestingly contained Rep20 and the plasmid sequences showed high nucleotide identity to each other, but possibly segregated due to recombination events. Isolate H1604 was the only strain containing the RepUS5 replicon plasmid.

Virulence genes were detected by nucleotide alignment to known genes. Twenty-five virulence factor genes were identified with more than 95% identity to known virulence factors (Supplementary Figure 2). None of the isolates carried both the *lukS-PV* and *lukF-PV* genes that code for Panton Valentine leukocidin (PVL), but the four isolates belonging to CC2596 and the single isolate belonging to CC2854 carried *lukF-PV* homolog genes (Supplementary Figure 2). All *S. argenteus* had *esxA, essC, adsA*, and *essA*. More than half harbored *sak, scn*, or *sasG*. The *scn* of the gorilla isolate showed 94% identity with the *S. aureus scn* (YP_001332910) whereas the *scn* from human *S. argenteus* isolates had an identity of more than 99.6%. The CC2596 isolates also carried *cna, cap5K, cap5I*, and *cap5J*. Furthermore, there were sporadic occurrences of *seb, ebh, esaB, seh, chp, lip, seg, esaC, esxB, sei, selu2, selm*, and *selo*. We identified known *S. aureus* adherence gene homolog where the identities were ranging from 76 to 99% with a mean of 87% (Supplementary Figure 3).

We found eight different bacteriophages in the genomes sequenced in this study and recombination events were shown between the bacteriophages (Supplementary Figure 4). Using homology searches it was evident that these bacteriophages resembled already known *S. argenteus* bacteriophages and *S. aureus* bacteriophages.

Using a BLASTn search a contig from isolate D7903 was identified which harbored a region of 13,477 bp having high identity to the pathogenicity island SaPIIshikawa11 of *S. aureus*. The region, which contained five nucleotide differences, had the typical features of SaPIs. It had the basic open reading frames of SaPIs with homology to *int, stl, str, xis, rep, pif*, and *ter*, which are responsible for their mobility and the region was flanked by two 15-bp direct repeats. In addition, the region harbored *seb* and *ear* genes. According to the nomenclature for pathogenicity islands in *Staphylococcus* species, we designated this genomic island SargPID7903 (Novick and Subedi, 2007). A gene synteny plot of SargPID7903 and SaPIIshikawa11 shows the high identity across the complete sequence (Supplementary Figure 5).

On another contig from isolate M4143, a partial sequence was identified showing high similarity (97%) to the first 12,363 bp of a second *S. aureus* pathogenicity island; SaPITokyo11212, which is 16,725 bp in size.

### Patient data

The 17 patients with MRSArg were originally all believed to be MRSA positive. Five patients presented with a skin and soft tissue infection (SSTI), while the remaining 12 patients were carriers. The carriers were MRSA screened for different reasons: Five due to presumed MRSA in the household, two had been hospitalized abroad, two were control swabbed following decolonization therapy due to previously finding of MRSA (t019/ST30 and t304/ST6), one had recurrent tonsillitis, one often traveled to the Philippines and one was au-pair in a family with MRSA. Seven of the patients had a known travel history to the Philippines (4), Vietnam (1), Lithuania (1), and Somalia/Yemen (1). The eight patients with methicillin susceptible isolates were sampled from wounds (*N* = 3), the ear (*N* = 2), the nose (*N* = 2), and in one case no clinical information was available. There was no data on travels for these patients.

## Discussion

In this study 25 *S. argenteus* isolates were described, 17 came from a collection of more than 4,000 presumptive MRSA isolates in Denmark and eight were presumable PCR *nuc* negative MSSA. This low prevalence of methicillin resistant *S. argenteus* is comparable with the findings from Belgium (Argudín et al., 2016). In a recent study from Thailand 58 out of 311 methicillin susceptible presumed *S. aureus* isolates were found to be *S. argenteus* (Chantratita et al., 2016). As we only whole genome sequence presumed MRSA isolates, we have no data on the prevalence of *S. argenteus* in Denmark among isolates that are believed to be MSSA.

It has been proposed that *S. argenteus* comprises more than a single clonal complex (Holt et al., 2011) and in this study the *S. aureus* MLST scheme analysis clearly showed that there were seven clonal complexes of *S. argenteus* (CC75, CC1223, CC2198, CC2250, CC2596, and CC2854 and one untypeable; ST-unknown). This was confirmed by the genomic data, which showed that each clonal complex clustered in a phylogenetic tree. Four of the seven clonal complexes were found in Danish patients. The clonal complexes not found were CC75, CC2198 and the untypeable one, where the former is only described in Australia. The discovery of four different clonal complexes in Danish samples could be ascribed to the travel history of the patients. The phylogenetic analysis shows clear clustering of ST2250, which also seems to be the most prevalent type. This is in accordance with the studies from Thailand. Interestingly, we here present a draft genome of ST2854, which has only previously been described in patients from Thailand.

The core genome analysis clearly shows a large difference in gene composition between *S. argenteus* and *S. aureus* and these two species share ~33% of genes. The *S. argenteus* isolates do, not surprisingly, share most of their genes. The pan-genome was sufficient to discriminate between *S. aureus* and *S. argenteus*. The antibiotic resistance pattern confirms that all the Copenhagen isolates are methicillin resistant and they all carry SCC*mec* type IV. The PCR *nuc* negative isolates from Odense have previously been proposed to be MSSA isolates (Hoegh et al., 2014), but we here classify them as *S. argenteus*. *S. aureus* isolates have a *nuc* gene length of 687 bp and the *nuc* gene is often not found by standard PCR in the isolates of *S. argenteus*. However, Tong et al. (2015) found that the *nuc* gene was amplified by PCR in *S. argenteus*, but found mismatches in the primer sites. In our study, a *S. argenteus nuc* gene was identified by WGS to have a length of 670 bp with 83% identity with the *S. aureus nuc* gene. A PCR with primers specific for the *S. argenteus nuc* gene could be developed for fast identification. MALDI-TOF identification of the isolates suggested *S. aureus* with a score between 1.7 and 2.0 in most cases. This has also been described by others (Tong et al., 2015) and is not unexpected due to the relatedness between the two species and the fact that *S. argenteus* was not included in the database. Including *S. argenteus* profiles in the database will improve the identification and give a much better idea of the prevalence and clinical significance of *S. argenteus*.

It has been speculated whether it is of clinical importance to differentiate between *S. aureus* and *S. argenteus*. A study (Chantratita et al., 2016) compared community-onset sepsis of *S. aureus* and *S. argenteus* and found some clinical differences between the two species mainly regarding respiratory failure. They also found fewer virulence factors in the *S. argenteus* isolates and PVL was only present in 16% of isolates compared to 51% of the *S. aureus*. In our study, none of the *S. argenteus* isolates carried both the *lukS-PV* and *lukF-PV* genes that code for PVL. However, five isolates carried the *lukF-PV*genes, but we have no data on the expression of these genes. The genes *esxA, essC, adsA*, and *essA* are part of the type VII secretion system in *S. aureus* and were present in all *S. argenteus* in this study and could be considered as part of the core genome of *S. argenteus*. Some isolates also had *esxB* and this combination is proposed to induce *S. aureus* pathogenesis of abscess (Burts et al., 2005). The *scn* gene was located on bacteriophages (Supplementary Figure 4) and this gene is known to be part of the human evasion complex in *S. aureus* ST398 and is not found in livestock (Stegger et al., 2013), and is therefore here of importance if the same differentiation is possible for *S. argenteus*. The *scn* is present in several of the clinical cases. It can therefore be hypothesized that *scn* has the same role in *S. argenteus*, and also that there could be an animal reservoir that is not yet discovered and that the *S. argenteus* lacking *scn* comes from this reservoir. The more distant gorilla *S. argenteus scn* gene suggests that there is a function of this gene in primate infections. The bacteriophages in *S. argenteus* seem to some extent to recombine which could have an effect on how *scn* is spread between isolates. The adherence genes of *S. argenteus* are most likely distant relatives to those of *S. aureus*. This could suggest a different function in *S. argenteus*. The plasmid replicon gene Rep16 was discovered in *S. aureus* and the plasmid is known to contain *BlaZ* and *CadDX* genes (McCarthy and Lindsay, 2012), but in this study it is shown to be prevalent in *S. argenteus*. These plasmids share high identity with known *S. aureus* plasmids suggesting interspecies transfer. There is a *CadD* and *CadC* gene in more than half of both *S. aureus* and *S. argenteus*. *CadC* hence, might not be a core gene, but is very prevalent and has been shown to increase Cadmium resistance (Massidda et al., 2006). The role of these plasmids and the relationship to *S. aureus* could be investigated further to identify the spreading potential of these plasmids.

In one isolate (D7903) of *S. argenteus* we identified a region with high similarity to the *S. aureus* pathogenicity island SaPIIshikawa11 (Sato'o et al., 2013). The region shared the typical features of *S. aureus* pathogenicity islands. The region had direct repeats at both ends and the genes responsible for the transfer of SaPIs, such as *int, rep*, and *ter* (Novick et al., 2010). Further, the region harbored the staphylococcal enterotoxin B gene, *seb*, and the penicillin-binding protein, *ear*. We propose, according to the nomenclature of phage-related chromosomal islands suggested by Novick et al. (2010), to designate this genomic island SargPID7903. The SaPI-like elements are very common in *S. aureus*, and have also been identified in other species, e.g., *S. haemolyticus* and *S. saprophyticus* (Kuroda et al., 2005; Takeuchi et al., 2005; Novick and Subedi, 2007). To the best of our knowledge, this is the first work that reports the presence of a SaPI-like element in a *S. argenteus* isolate. The presence of the *seb* gene on SargPID7903 can potentially lead to increased virulence of *S. argenteus*, as has been indicated for *S. aureus* strains carrying *seb*-harboring SaPIs (Sato'o et al., 2013).

In general, *S. argenteus* has many similarities with *S. aureus*, but the differences became apparent, when comparing the core genome of the two species. There is a large gene pool that is not overlapping between the two species and they should be considered as two independent species that can share genomic material such as, SaPIs, plasmids, and bacteriophages. In countries with contact isolation of MRSA positive patients and eradication regimens for MRSA carriers, a low amount (<1%) of identified MRSA will be *S. argenteus* leading to unnecessary isolation and eradication therapy. Further studies of the prevalence in the Far East and future identification by MALDI-TOF, will determine the clinical relevance of *S. argenteus*.

## Author contributions

The authors all gave substantial contributions to the conception or design of the work; or the acquisition, analysis, or interpretation of data for the work. All authors all worked on drafting the work or revising it critically for important intellectual content and gave a final approval of the version to be published. All authors agree to be accountable for all aspects of the work in ensuring that questions related to the accuracy or integrity of any part of the work are appropriately investigated and resolved.

### Conflict of interest statement

The authors declare that the research was conducted in the absence of any commercial or financial relationships that could be construed as a potential conflict of interest.
