# Supplementary material for: Whole Genome Sequencing of Danish Staphylococcus argenteus Reveals a Genetically Diverse Collection with Clear Separation from Staphylococcus aureus
Source: Front Microbiol. 2017 Aug 9;8:1512. doi: 10.3389/fmicb.2017.01512 (PMC5552656; doi:10.3389/fmicb.2017.01512)
Supplement: Supplementary file 2 [file DataSheet1.docx]

Supplemenatry Figure 1. Principal component analysis of a hierarchical clustering of the presence and absent of genes in 35 *S. argenteus* and 22 *S. aureus*. The analysis were run on 4 different gene homology settings (85, 90, 95, 100 %). Purple squares are *S. argenteus* and orange is *S. aureus*. PC1 and PC2 are principal component 1 and principal component 2 respectively.
