# Supplementary material for: Whole Genome Sequencing of Danish Staphylococcus argenteus Reveals a Genetically Diverse Collection with Clear Separation from Staphylococcus aureus
Source: Front Microbiol. 2017 Aug 9;8:1512. doi: 10.3389/fmicb.2017.01512 (PMC5552656; doi:10.3389/fmicb.2017.01512)
Supplement: Supplementary file 4 [file DataSheet3.docx]

Supplementary Figure 3. Heatmap of identity of virulence genes found in each *S. argenteus* isolate with identity larger than 70% to known adherence genes. The red color indicates high identity and the blue color indicate lower identity. Isolates prefixed with Sa_ are *S. argenteus* genomes downloaded from NCBI, the isolate prefixed with SA_ is a *S. aureus* and the rest are Danish isolates from our database.
