# Supplementary material for: Whole Genome Sequencing of Danish Staphylococcus argenteus Reveals a Genetically Diverse Collection with Clear Separation from Staphylococcus aureus
Source: Front Microbiol. 2017 Aug 9;8:1512. doi: 10.3389/fmicb.2017.01512 (PMC5552656; doi:10.3389/fmicb.2017.01512)
Supplement: Supplementary file 5 [file DataSheet4.docx]

Supplementary Figure 4. Comparison of the eight *S. argenteus* phages discovered using Phaster. There are signs of recombination between number 2, 3 and 4 phages from the top.
