# Supplementary material for: Whole Genome Sequencing of Danish Staphylococcus argenteus Reveals a Genetically Diverse Collection with Clear Separation from Staphylococcus aureus
Source: Front Microbiol. 2017 Aug 9;8:1512. doi: 10.3389/fmicb.2017.01512 (PMC5552656; doi:10.3389/fmicb.2017.01512)
Supplement: Supplementary file 6 [file DataSheet5.docx]

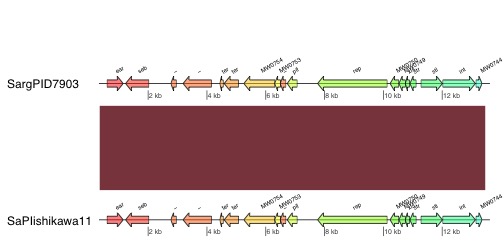


Supplementary Figure 5. Gene synteny plot of SargPID7903 and SaPIishikawa11. The high nucleotide identity >99 % between SargPID7903 and SaPIishikawa11 is visualised.
